# Monte Carlo modeling in CT-based geometries: dosimetry for biological modeling experiments with particle beam radiation

**DOI:** 10.1093/jrr/rrt118

**Published:** 2013-12-05

**Authors:** Eric S. Diffenderfer, Derek Dolney, Maximilian Schaettler, Jenine K. Sanzari, James Mcdonough, Keith A. Cengel

**Affiliations:** Department of Radiation Oncology, University of Pennsylvania, 3400 Civic Center Blvd, 8-136 SCTR Philadelphia, PA 19104, USA

**Keywords:** solar particle event, Monte Carlo, dosimetry, animal models, radiobiology, inhomogeneous dose distribution, stopping beams

## Abstract

The space radiation environment imposes increased dangers of exposure to ionizing radiation, particularly during a solar particle event (SPE). These events consist primarily of low energy protons that produce a highly inhomogeneous dose distribution. Due to this inherent dose heterogeneity, experiments designed to investigate the radiobiological effects of SPE radiation present difficulties in evaluating and interpreting dose to sensitive organs. To address this challenge, we used the Geant4 Monte Carlo simulation framework to develop dosimetry software that uses computed tomography (CT) images and provides radiation transport simulations incorporating all relevant physical interaction processes. We found that this simulation accurately predicts measured data in phantoms and can be applied to model dose in radiobiological experiments with animal models exposed to charged particle (electron and proton) beams. This study clearly demonstrates the value of Monte Carlo radiation transport methods for two critically interrelated uses: (i) determining the overall dose distribution and dose levels to specific organ systems for animal experiments with SPE-like radiation, and (ii) interpreting the effect of random and systematic variations in experimental variables (e.g. animal movement during long exposures) on the dose distributions and consequent biological effects from SPE-like radiation exposure. The software developed and validated in this study represents a critically important new tool that allows integration of computational and biological modeling for evaluating the biological outcomes of exposures to inhomogeneous SPE-like radiation dose distributions, and has potential applications for other environmental and therapeutic exposure simulations.

## INTRODUCTION

The space radiation environment imposes dangers of acute and chronic exposure to ionizing radiation, especially during space travel missions outside the protection afforded by the Earth's magnetosphere. Exposure to ionizing radiation from a solar particle event (SPE) is of particular concern. These events are difficult to forecast and consist primarily of protons with kinetic energies ranging from 10 MeV up to several GeV [1, 2]. Due to the low energy of most incoming protons, we expect a highly inhomogeneous dose distribution, with the majority of the dose being delivered to superficial tissue. The acute radiobiological effects of low-dose proton radiation are not well understood and are further complicated by the inhomogeneous distribution of the dose to sensitive organs.

The Center for Acute Radiation Research (CARR) is evaluating the effects of SPE-like radiation dose distributions in biological systems to better understand the potential spectrum of acute toxicity of whole body, inhomogeneous proton radiation exposure with skin doses that far exceed internal doses [3]. This work is being performed using whole-body exposures to proton radiation with SPE-like energy/fluence profiles in multiple animal models, including mice [4], Yucatan minipigs [5, 6], and ferrets [7] to evaluate toxicity with a systems-based approach. Conventional techniques for correlating dose with toxicity rely on gamma rays as a reference, but this comparison is of limited use here due to the extreme differences in dose distribution between gamma rays and SPE-like proton radiation. Thus, the CARR team has also sought to determine the relative biological effectiveness (RBE) of SPE proton radiation by comparing SPE-like proton exposures with electron radiation exposures with a similar macroscopic organ dose distribution [8]. In that study, a combination of 6 MeV and 12 MeV electrons was shown to be more effective at reproducing the dose distribution from SPE-like proton radiation than conventional gamma rays.

Before one can begin to relate the dose to toxicity, it is essential to fully understand the dose distribution within the experimental animals at the time of exposure. Previous dosimetry experiments primarily consisted of point dose measurements using calibrated spherical, thimble, or parallel plate type ionization chambers, such as the system employed at the NASA Space Radiation Laboratory [9]. However, extrapolating these point dose measurements to a full-body dose for SPE-like proton or electron radiation is made difficult through the inhomogeneous dose distributions. Previously, we have applied conventional treatment-planning techniques using commercially available dosimetry software [5, 8], but the software is not well suited to analyzing total-body proton radiation for non-human animals in variable geometries. A need exists for software that can accurately calculate doses delivered to a variety of geometries.

To that end, we sought to develop a dosimetry package that will provide spatially resolved dosimetry information in CT-based geometries using the Geant4 Monte Carlo (MC) simulation framework [10]. We validated the package using data from experiments with proton SPE and electron simulated SPE exposures [5, 6, 8]. The software can help determine the sensitivity of dosimetric quantities to numerous physical parameters such as energy spectrum, shielding, radiation source geometry, and anatomical variation.

## MATERIALS AND METHODS

Monte Carlo (MC) simulations were developed using the Geant4 C ++ programming toolkit ver. 9.4.p2 [10] to investigate dose distributions for radiobiology experiments using simulated proton SPE beams at Loma Linda University Medical Center (LLUMC) and large field electron beams at the University of Pennsylvania Department of Radiation Oncology (UPENN). The Geant4 physics list we used was based on one reported to give good agreement with proton depth–dose measurements in water and multilayer Faraday cup measurements [11]. As recommended by the authors of that study, the Geant4 standard electromagnetic physics models, the UHElastic nuclear elastic scattering model, and the binary cascade nuclear inelastic model was used. However, newer versions of Geant4 released after that paper was published require that different multiple Coulomb scattering models be used for electrons/positrons and for hadrons/ions. Because of that, we used G4eMultipleScattering for the electrons/positrons and G4hMultipleScattering for the hadrons. These new models should be more accurate since the scattering model parameters have been fitted separately for the different kinds of particles. Simulations modeling the Roberts Proton Therapy Center beam line at UPENN using these physics models have been compared with water phantom measurements with ion chambers and film in an unpublished study. The details of modeling the UPENN proton beam line is beyond the scope of the current study, but the use of the same physics list to model the proton beam at LLUMC is justified on the grounds that the simulations of the UPENN proton beam range agree to within 2 mm, and the dose agrees to within 2%, even in the penumbra, for both collimated and scanned proton beam simulations. For the electron beam simulations, along with G4eMultipleScattering, we have also used the low-energy electromagnetic physics processes (G4LowEnergyIonisation and G4LowEnergyBremsstrahlung) for electrons. For photons generated in the electron beam simulations we used G4LowEnergyPhotoElectric, G4LowEnergyCompton, G4LowEnergyGammaConversion, and G4LowEnergyRayleigh physics processes. The introduction of the low energy processes in our simulation was necessary to reproduce the measured electron depth dose profiles that are described below.

**Fig. 1.**
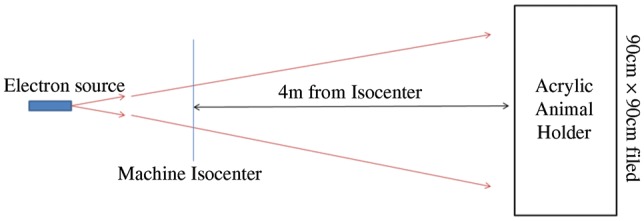
A schematic diagram depicting the electron beam geometry used in radiobiological experiments and in MC simulations to determine the dose distributions. The animal enclosure is placed 4 m downstream from the machine isocenter located at 1 m downstream from the electron source. The machine isocenter is precisely defined for therapeutic linear accelerators such as the Varian 2100iX used in this study.

In the electron beam animal experiments modeled here, an electron field approximating the dose distributions expected from an SPE was created using a Varian Clinac 2100 iX (Varian Medical Systems) clinical linear accelerator located at UPENN. The field consists of a weighted combination of beams with nominal energies of 6 and 12 MeV [8]. To achieve the large field size (90 cm × 90 cm) needed for animal irradiation, a 5 m source-to-surface distance (SSD) was used with the *x*- and *y*-collimating jaws of the accelerator set to define a field size of 34 cm × 34 cm when measured at the machine isocenter. Figure 1 provides a schematic view of the electron beam irradiation geometry.

The doses delivered by each electron beam are defined at the depth of maximum dose along the depth profile (d_max_). The combined 6 + 12 MeV electron beam simulating an SPE dose distribution is formed with 80% of the prescribed dose as 6 MeV electrons and 20% as 12 MeV electrons. Depth dose profiles for the 6 and 12 MeV electron beams were measured under the beam geometry of Fig. 1 using a Scanditronix (IBA Dosimetry) electron diode EFD-3G (S/N 4095) in a layered solid water plastic phantom (Gammex, Inc.). To verify beam uniformity in the horizontal plane, depth profile measurements were taken at 15.2, 30.5, 45.2 and 61.0 cm off axis. Additional measurements verifying the surface dose uniformity were performed along the vertical axes.

The Monte Carlo simulation of the electron beam from the Varian Clinac at UPENN was modeled as a circular planar (2 cm radius) source at 1 m upstream from the machine isocenter in a 2 m × 2 m × 6 m air-filled volume. The electron beam energies produced by the Clinac were determined to be 6.02 MeV and 12.23 MeV using measurements obtained during machine commissioning and the protocol for most probable energy [12]. A 10% Gaussian spread was applied to the source energy in simulation. The beam was clipped at isocenter to a 34 cm × 34 cm field size. A voxelized 20 cm × 20 cm × 20 cm volume (1 mm^3^ voxels) was placed within the simulation at an SSD of 5 m. The simulation material of the voxelized phantom was defined (8.09% H, 67.17% C, 2.41% N, 19.88% O, 0.14% Cl, 2.31% Ca) to match the elemental composition of Solid Water (Gammex, Inc.). Dose was scored in the voxelized phantom for 2 × 10^8^ histories for both electron energies, and depth profiles were constructed by integrating within a 5 mm radius along the beam axis. Additionally, off-axis profiles were produced by shifting the central axis of the phantom laterally to match the measurement points discussed above.

A simulation of the proton SPE beam at LLUMC was developed by fitting MC-generated pristine Bragg peaks to the depth dose data described in Sanzari *et al*. [6]. The depth dose data alone was insufficient to build a Monte Carlo model of the proton beam; detailed information on the proton energy spectrum was required. Furthermore, information on the source geometry was limited to our knowledge of the size and uniformity of the radiation field at the animal irradiation position, which is where beam verification measurements were reported. Therefore, since the LLUMC proton beam showed high uniformity over an area larger than was required for the animal irradiations, the proton beam was modeled as a broad parallel beam. To approximate the energy spectrum that would produce a depth dose profile matching the measurements, our model was composed of a weighted sum of Bragg peaks with energies from 20–80 MeV in 1 MeV increments. Bragg peaks for each energy were generated in the MC code and scored for 10^7^ histories in a voxelized 10 cm × 10 cm × 10 cm volume (0.5 mm^3^ voxels) volume composed of the predefined Geant4 material G4_WATER. Depth dose profiles were constructed by integrating within a 5 mm radius along the beam axis. Bragg peak weights were calculated with a least squares non-negative fit to the LLUMC depth dose data using the mathematical analysis software Matlab (The Mathworks, Inc.). A proton spectrum file was created from the Bragg peak weights and a broad parallel beam was generated and used to irradiate the CT geometry described below.

To model the dose distributions to animal subjects during proton and electron SPE experiments, the MC simulations incorporated voxelized geometries based on CT scans. The CT images are 3D matrices of Hounsfield units (HU), which are a measure of electron density within each voxel relative to water. The image files, along with a structure file containing a 'BODY' contour that defines the edges of the body for each image slice within the rectangular HU array, were imported into the simulation in Digital Imaging and Communications in Medicine (DICOM) format. A voxelized geometry was built using tissue materials to replace the HU of each voxel that lies within the body contour. The procedure for assigning materials to the voxels, which span thousands of HU, followed that of Jiang and Paganetti [13], who assigned tissue compositions to ranges of HU based on analysis of CT images and varied the material density within those ranges. The Geant4 predefined material representing the NIST standardized definition of air (G4_AIR) was placed in the voxels that lie outside of the body. To conserve memory, the voxelized geometry was clipped to the limits defined by the BODY contour, with the remaining empty volume of the simulation geometry consisting of G4_AIR.

The dose deposited within each voxel was accumulated event-by-event and written to a dose matrix file. Total simulation time for 10^7^ event histories can take up to 15 h on a 2.3 GHz processor with 4 GB of RAM, depending on the primary particle identity (electron or proton), the primary beam energy, and the number of voxels. Statistical accuracy in MC simulations depends on the total number of event histories and can be evaluated by calculating standard deviations of mean dose to organs of interest over multiple batches of event histories. This work serves to demonstrate the benefits of Monte Carlo dose simulations for radiobiology experiments, and quantifying statistical accuracy of dose to specific organs will need to be performed on a case-by-case basis. However, with field sizes exceeding 1 m^2^ at the point of intersection with the CT geometry, we have found that 10^8^–10^9^ event histories are necessary to minimize the appearance of isodose line fluctuations in the dose distributions. To limit computation times, we have run our simulations in batches of 10^7^ events on a 116 CPU cluster. Each batch is initialized with a different seed number and the files containing the simulation results are combined. The cumulative files are converted to the DICOM format, allowing the dose distributions to be imported into commercially available visualization software.

MC simulation of animal irradiations included the effects of the enclosure used during the experiments by placing the CT geometry within a rectilinear PMMA enclosure with 5 mm thick walls. A 10 cm air gap was maintained between the inner wall of the animal enclosure and the edge of the CT geometry, which coincides with a box bounding the edges of the CT BODY contour. The animals in the biological experiments were free to move throughout the duration of the actual exposures, which leads to variations in the air gap between the inner surface of the enclosure and the surface of the animal. The dose variation was simulated for the 6 MeV electron beam experiments by measuring the dose profile in the voxelized Solid Water phantom, as described above, but with the addition of a 5 mm thick PMMA wall and for air gaps of 0, 1, 3, 5, 10, 30, 50 and 100 mm between the wall and the phantom. Among the beams used in these simulations, 6 MeV electrons will exhibit the highest proportion of lateral scattering, which is directly linked to any observed difference in depth dose profile after traversing a variable length air gap.

Animal motion during electron or proton irradiation also introduced a source of variation through changes in the incident beam angle relative to the animal's body axis. The animal enclosures were oriented such that the long (crown–rump) axis was perpendicular to the beam direction. During the experiments simulated here, the enclosures were rotated 180^o^ at even intervals throughout the irradiations to ensure uniform dose distributions over the entire animal body. Even so, the animals were observed to shift position and orientation with respect to the beam. To incorporate this motion into the MC simulations, the electron beam experiments were recorded on video, and the beam angle in the simulation can be varied between the four cardinal angles (0°, 90°, 180° and 270°) relative to the long axis and combined in a weighted sum to account for the average position of the irradiation subject. To investigate the effects of animal orientation on the dose distributions, we performed the simulations with beam angles of evenly weighted opposed lateral beams (beam angles 0° and 180^o^), evenly weighted opposed anterior–posterior beams (beam angles 90° and 270°), and a weighted combination of 70% opposed lateral with 30% opposed anterior–posterior beams.

Another factor adding to dosimetric uncertainty for these types of irradiation experiments is variation in animal size. This is especially pronounced for large animals such as the Yucatan minipigs, which arrive from the vendor with significant batch size variations and grow considerably during the time leading up to the irradiation phase of the experiment. Due to logistical constraints, a consistent size cannot always be maintained from experimental cohort to cohort. This situation occurred during the LLUMC proton SPE experiment [6], when the pigs were noticeably smaller than the animals used in the electron beam experiments. We have taken account of this situation by using CT scans of a relatively small and a relatively large animal in the proton and electron beam simulations, respectively. The small animal has a crown–rump length of ∼ 54 cm, and the large animal has a crown–rump length of ∼ 63 cm.

For the purpose of comparing irradiation conditions, bone marrow (BFO), skin, eyes, and lungs were segmented in each CT image using Eclipse ver. 10 (Varian Medical Systems) and imported into MIM ver. 5.2 (MIM Software, Inc.) visualization and analysis software, along with the dose distribution files produced in simulation. Dose–volume histograms (DVHs) for the segmented organs were created for the electron beam and proton SPE exposure scenarios.

## RESULTS

**Fig. 2.**
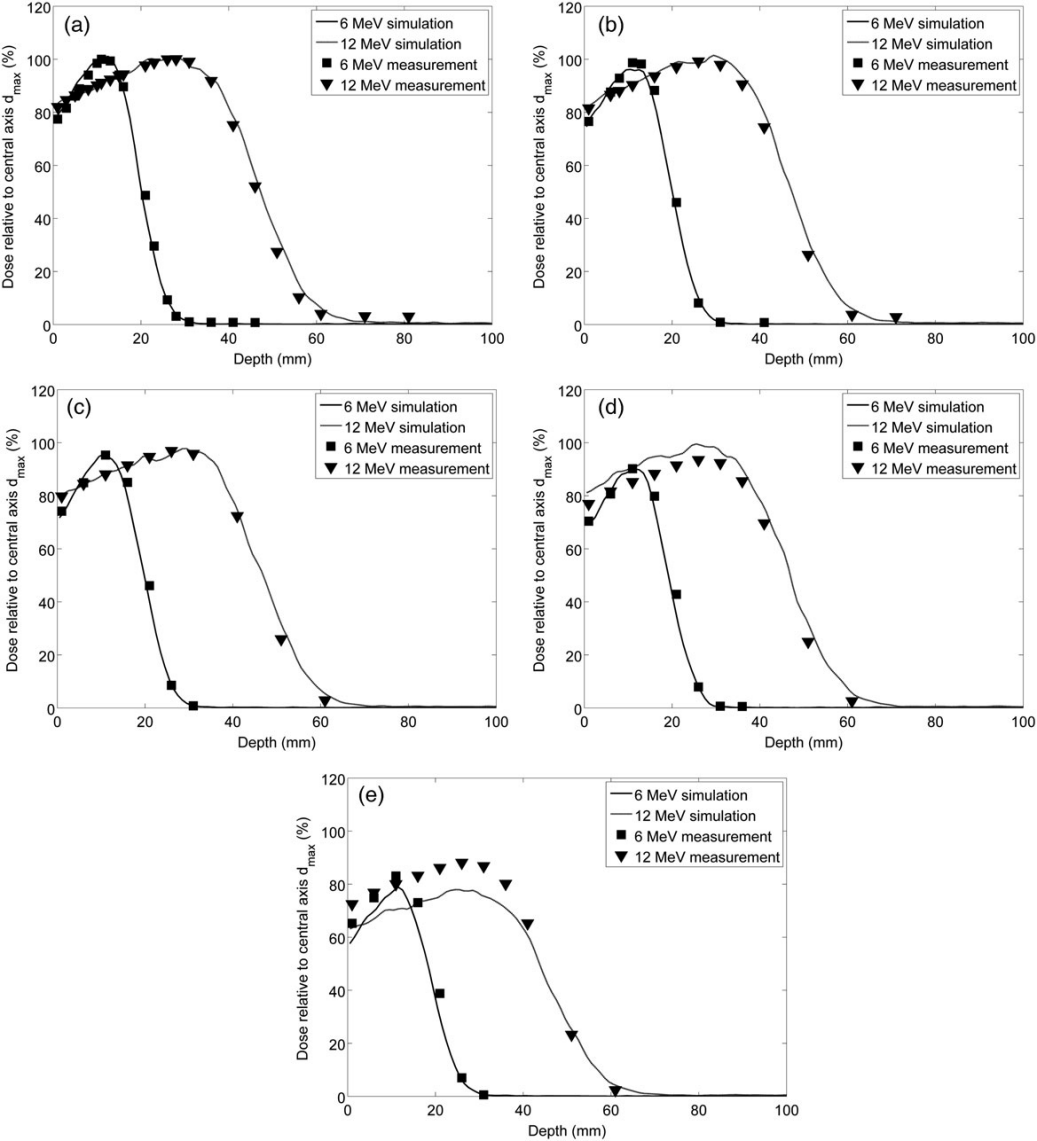
Diode measurements in solid water and MC simulated depth dose profiles on the (**a**) central axis (CAX), and at lateral shifts of (**b**) 15.2 cm, (**c**) 30.5 cm, (**d**) 45.2 cm, and (**e**) 61.0 cm. The depth dose profiles are shown relative to the maximum dose on CAX for the 6 MeV (squares) and 12 MeV (triangles) electron beams.

To validate the Geant4-based MC modeling of the electron beam, we have compared predicted versus measured depth dose profiles using a rectangular solid water phantom and matching beam geometry as in Fig. 1. This comparison demonstrates that the MC accurately reproduces measured dose along the central axis (CAX) of the beam for both 6 MeV and 12 MeV electron beams (Fig. 2a). The MC simulation matches the measured profiles to better than 3% at d_max_ out to a lateral distance of 30.5 cm (Fig. 2b–c), however the model begins to fail with differences exceeding 5% when the profiles are examined further off-axis between 45.2 cm and 61 cm (Fig. 2d–e). This discrepancy likely results from the simplifying assumptions made in the modeling of the equipment used to generate the electron beam, but it has only a minor effect on the simulation results, since the field area for animal irradiation was confined to a radius of < 45 cm.

**Fig. 3.**
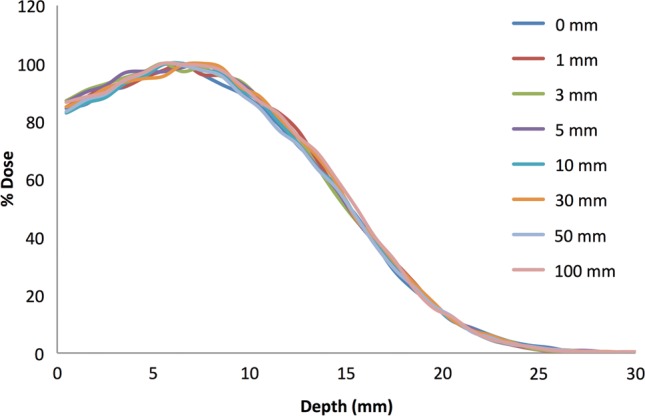
MC simulated depth dose profiles of the 6 MeV electron beam with a 5 mm PMMA wall and air gaps of 0, 1, 3, 5, 10, 30, 50 and 100 mm.

Because the experimental setup of the radiobiology experiments that were simulated allowed for animal movement within the confines of the animal enclosure during irradiation, the air gap between the enclosure surface and the surface of the animal varied by up to 10 cm. Therefore, we used the MC electron beam model to determine the effects of this variable air gap on the depth dose profile. The results of these simulations demonstrate that there is very little variation with air gap and no clear trend in the minor statistical variations (2% at d_max_) within the depth dose profiles (Fig. 3). This suggests that any variability of dose distribution within the animal due to variation in air gap, caused by the animal motion during irradiation, is minimal and unlikely to confound the biological data obtained in such experiments.

**Fig. 4.**
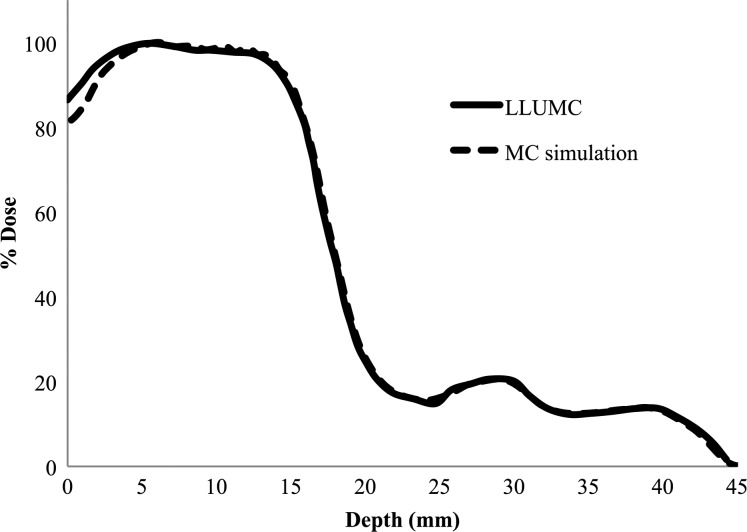
MC simulated depth dose profile produced by a weighted sum of pristine proton Bragg peaks fit to the depth dose profile of the proton beam produced at Loma Linda University Medical Center.

Shown in Fig. 4 is the measured and simulated depth dose profile of the LLUMC proton SPE beam with a 5 mm shift to account for the plastic animal enclosure wall. The measurement and simulation match well with the exception of a maximum 6% difference in the first 5 mm of depth after the enclosure wall. Differences may be due to scattered low-energy protons not accounted for in our fit to the measured data, which used a weighted sum of pristine Bragg peaks. The impact primarily affects our estimation of the skin dose in the proton irradiation experiments. However, the dose to the skin and organs at depth is accurate within the uncertainties inherent in biological experiments of this scale. Further refinement of the LLUMC proton beam model was deemed unnecessary and beyond the scope of this study.

**Fig. 5.**
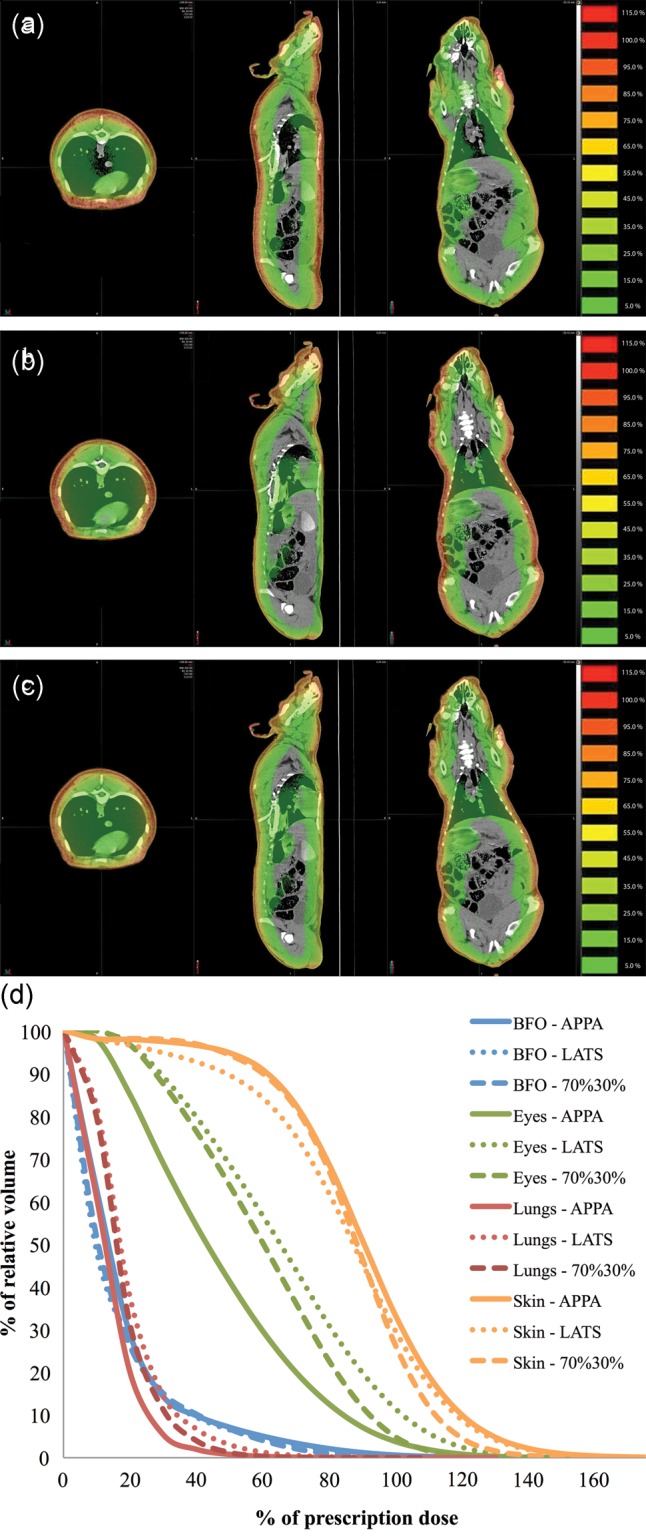
Dose distributions overlaid on Yucatan minipig CT images for 6 + 12 MeV electron irradiations for (**a**) opposed anterior–posterior beams (APPA), (**b**) evenly weighted lateral opposed (LATS), and (**c**) a combination of 70% LATS with 30% APPA beams. The images shown are the transverse, sagittal and coronal planes. (**d**) DVHs of eyes, lungs, BFO and skin for the three beam orientations.

In the next series of simulations, dose distributions were determined for geometries constructed from CT images of Yucatan minipigs used during proton SPE and 6 + 12 MeV electron irradiations. These datasets were transferred to MIM visualization software for analysis. This software package is used clinically by radiation oncologists to visualize dose distributions in order to predict the relative toxicity and efficacy for radiation treatments of human patients. Rotational movement of the animals about the long (crown–rump) axis was approximated in the simulations by weighting the beam angles differently before summing to create the final dose distribution. All of the animal irradiation procedures that we have simulated were recorded at the time of the experiments, and videos of specific animals can be analyzed in the future to determine the relative percentage of time each animal spent in different orientations relative to the beam. Thus, these simulations allow determination of the magnitude of interanimal variability in dose distribution that is produced by freedom of animal movement, which is made necessary by low dose rates and long irradiation periods (typically 3–5 h). For example, an animal that lies in a right (left) lateral decubitus position during the entire irradiation would receive a dose from the dorsal/ventral sides, as demonstrated in Fig. 5a, which depicts the dose distribution in the transverse, sagittal and coronal planes for evenly weighted opposed anterior–posterior (APPA) beams (beam angles 0° and 180°). In contrast, an animal that stays entirely in the desired prone orientation would have equal doses from each side, as demonstrated in Fig. 5b, which provides the same views for evenly weighted opposed lateral (LATS) beams (beam angles 90° and 270°). In practice, the animals alternate between prone and right (left) lateral decubitus orientation, such that the relative contribution can be approximated with a weighted combination of 70% prone with 30% right (left) lateral decubitus (Fig. 5c). Figure 5d displays DVHs of eyes, lungs, BFO and skin for the three irradiation configurations presented in Fig. 5a–c. Doses in Fig. 5a–d were calculated with the electron beam using the large Yucatan minipig CT dataset. Clearly, animal motion and relative orientation with respect to the beam axis contributes to a noticeable difference in dose distribution.

**Fig. 6.**
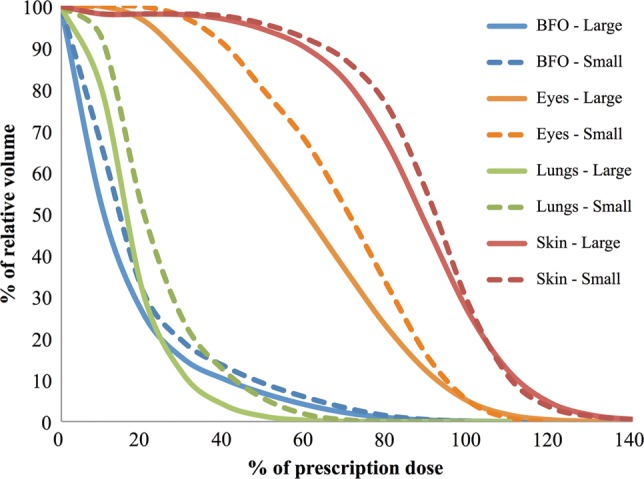
DVHs of eyes, lungs, BFO and skin are compared for the large- and small-sized animal CT datasets for the electron beam with a 70% LATS and 30% APPA beam orientation.

Another potential experimental variable that can be difficult to control is animal size, especially when using a large animal model such as pigs. In experiments comparing the effects of proton and electron simulated SPE exposures, due to variations in animal size from the vendor and scheduling restrictions inherent to animal experiments using proton exposures, we cannot always use animals of the exact same mass for all experiments. This is critical in the use of ‘stopping beams’ where the depth of specific organs and consequent organ dose distribution will change depending on the animal size. To estimate the effects of animal size on the organ dose distribution for animals exposed to proton and electron SPE, we have CT datasets for Yucatan minipigs of two different sizes and have used these in our computational dose modeling. Figure 6 presents DVHs of dose to eyes, lungs, BFO and skin using the electron beam with a 70% LATS plus 30% APPA configuration in the large and small Yucatan minipig CT dataset. Even though the animal size difference between the two CT datasets is just 9 cm crown–rump, it is evident that size must be factored into dosimetry computations involving SPE-like radiation.

**Fig. 7.**
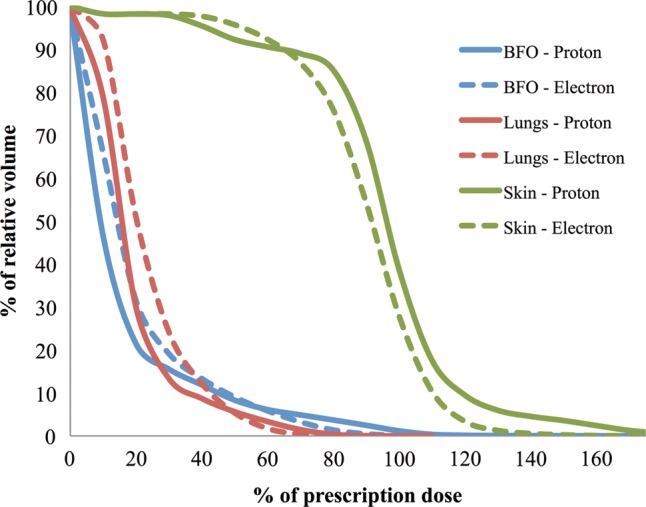
DVHs of BFO, skin and lungs are compared for electron and proton simulated SPEs that account for differences in animal size between electron and proton exposure experiments.

These studies have allowed us to systematically compare the macroscopic dose distribution between electron and proton simulated SPE exposures, and to understand the consequent subtle differences in organ dosimetry. Figure 7 displays DVHs calculated for the large pig CT set that was irradiated with electrons compared with the small pig CT set irradiated with protons in the study of Sanzari *et al*. [6]. The dose distributions from the radiobiological experiments using two different radiation sources and with animals of different size exhibit clear differences in dose to vital organs, which should be considered when evaluating biological response to the separate experiments. These computational modeling studies are a critical component of interpreting the biological impact of dose heterogeneity between animal exposures, and have allowed us to improve our estimates of RBE for SPE exposures [5, 6].

## DISCUSSION

In this manuscript, we describe a Geant4-based MC modeling algorithm for CT datasets that has the demonstrated capability of calculating detailed dose distributions from radiation produced in radiobiology experiments. Our beam models have been verified with measured data, and they allow us to more accurately quantify the dose delivered to animals during radiobiology experiments that are designed to simulate an SPE exposure. These experiments are being conducted to determine the acute effects of this type of radiation, and with the large heterogeneity observed in charged particle dose distributions it is important that we accurately estimate the dose to sensitive organs.

In experimental setups, our simulation accurately models the electron depth dose profile for points with up to 45 cm lateral shift from the central beam axis for both 6 MeV and 12 MeV electron beams at UPENN (Fig. 2) and demonstrates that animal movement is not an issue for concern insofar as it affects the air gap maintained between the inner surface of the animal enclosure and the animal's skin (Fig. 3). We have modeled the depth dose profile produced by proton beam experiments performed at LLUMC and have incorporated this model into simulations for the purpose of comparing biological results obtained under different irradiation conditions. This modeling can also take into account differences in animal orientation observed during experiments (Fig. 5).

**Fig. 8.**
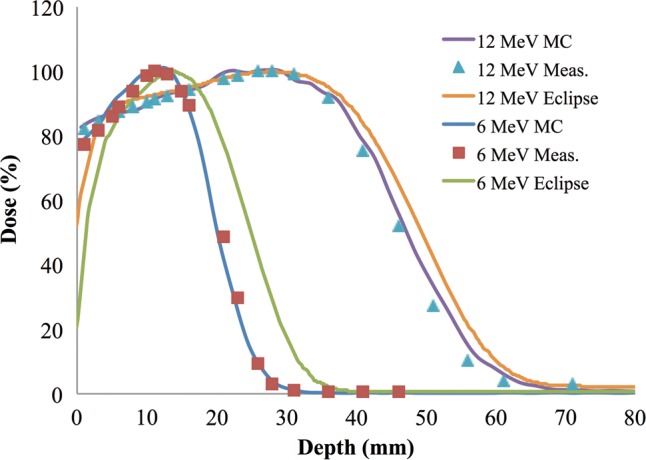
MC simulated depth dose profiles on the central axis (CAX) for the 6 MeV and 12 MeV electron beams using the current Geant4-based model and the previous [8] Eclipse eMC algorithm (Eclipse) as compared with diode measurements of 6 MeV (squares) and 12 MeV (triangles) in a layered solid water phantom.

Previously, we demonstrated results of MC-based simulation using commercial software designed for patient treatment [8]. One problem with using this software is that the beam modeling is not designed to be accurate when the treatment machine is operated outside the typical parameters for human use, as is the case with the animal experiments that are simulated here. These experiments use a geometry that is unlike the typical human electron treatment, which employs a much smaller source-to-skin distance and a treatment ‘electron cone’ that limits the beam size to no more than 20 cm × 20 cm at the surface of the skin. To illustrate this difference, we compared the simulation results [8] of the Eclipse eMC algorithm (Varian Medical Systems), our current Geant4-based model, and the measured data for the 6 MeV and 12 MeV electron beams in a layered solid water phantom. This showed that our current simulation matches the measured data relatively better than the Eclipse model, which displays discrepancies with the data under these model conditions, even along the central axis of the beam (Fig. 8).

Due to computational complexity, investigations of the absorbed dose to organs that would be expected from exposure to an SPE [3, 14–16] have been largely based on simplified models of radiation transport in matter that rely on a straight-ahead approximation or ray-tracing, and neglect the effects of the production of secondary radiation, multiple scattering, angular straggling, and material heterogeneity. Often, doses to individual organs are represented by dose absorbed at average depths in a standard sphere. Townsend and Zapp [15], Zapp *et al*. [17] and Bahadori *et al*. [18] have investigated the differences in organ doses due to organ self-shielding and body-size variation, using computerized anatomical models, and have found organ dose variations as large as 15% over standardized equivalent sphere models. Advances in computational power and computed tomography (CT) imaging techniques have enabled comprehensive models of individual human anatomy to be incorporated into radiation transport simulations that incorporate the full complement of radiation interaction processes including multiple scattering, nuclear interactions, and the production of secondary radiation.

One potential area that could further extend the use of our current modeling approach would be for personalized astronaut dosimetry. The extension of the MC software presented here to a model of astronaut SPE radiation exposure is a simple matter of incorporating appropriate CT and radiation source geometries into our existing MC software. In the event of SPE exposure, rapid assessment of the risk posed to astronauts will require accurate estimation of dose distributions for each astronaut. For higher energy particles contained in galactic cosmic rays, the individual variation in astronaut anatomy is unlikely to alter the dose distribution within each astronaut, and the interastronaut variability would therefore be small. However due to the lower but highly variable energy spectrum of SPE particles and the variables of shielding and angular distribution of particles, individual astronaut anatomy may contribute to variability in the resulting dose distribution. At the very least, understanding what the measured energy spectrum and fluence anisotropy of an ongoing SPE does to the dose distribution may be critical in prescribing counter measures and in adaptation of mission planning. With further development, the current model has the potential to provide rapid assessment of dose distributions and biological effects within individual astronauts for specific SPEs and to bring the potential promise of personalized radiation medicine into the space program.

## CONFLICT OF INTEREST

The authors declare that there are no conflicts of interest.

## FUNDING

This work was supported by the Center of Acute Radiation Research (CARR) grant from the National Space Biomedical Research Institute (NSBRI) through NASA NCC 9-58, NIH Training Grant 2T32CA00967, NIH CA-140116, and the US Army Medical Research and Materiel Command under Contract Agreement No. DAMD17-W81XWH-07-2-0121. Opinions, interpretations, conclusions and recommendations are those of the author and are not necessarily endorsed by the US Army.
